# Budd-Chiari Syndrome: Shunt or Transplant?

**DOI:** 10.1155/1998/69641

**Published:** 1998-12

**Authors:** Marshall J. Orloff

**Affiliations:** University of California, San Diego Medical Center 200 West Arbor Drive San Diego CA 92103-8999 USA


mosis. In our experience, roughly 10% of patients will have low volume amylase-rich fluid draining via the drains. Over 85% of these low volume pancreatic fistulas will heal with conservative management. While pancreatic fistula has not disappeared as a postoperative complication, it is certainly no longer the dreaded and feared complication that it was several decades ago. As additional experience and data are gathered, perhaps one particular technique of pancreatic reanastomosis will assume priority.

### *References*

[1] Funovics, J. M., Zoch, G., Wenzl, E. and Schulz, F. (1987). Progress in reconstruction after resection of the head of the pancreas. *Surg. Gynecol. Obstet.*, **164**, 545–548.

[2] Hiraoka, T., Kanemitsu, K., Tsuji, T. *et al.* (1993). A method for safe pancreaticojejunostomy. *Am. J. Surg.*, **165**, 270–272.

[3] Biehl, T. and Traverso, L. W. (1992). Is stenting necessary for a successful pancreatic anastomosis *Am. J. Surg.*, **163**, 530–532.

[4] Kingsnorth, A. N. (1989). Duct to mucosa isolated Roux loop pancreaticojejunostomy as an improved anastomosis after resection of the pancreas. *Surg. Gynecol. Obstet.*, **169**, 451–453.

[5] Kram, H. B., Clark, S. R., Ocampo, H. P., Yamaguchi, M. A. and Shoemaker, W. C. (1991). Fibrin glue sealing of pancreatic injuries, resections and anastomoses. *Am. J. Surg.*, **161**, 479–482.

[6] Matsumoto, Y., Fujii, H., Miura, K. *et al.* (1992). Successful pancreatojejunal anastomosis for pancreatoduodenectomy. *Surg. Gynecol. Obstet.*, **175**, 555–562.

[7] Greene, B. S., Loubeau, J. M., Peoples, J. B. and Elliott, D. W. (1991). Are pancreatoenteric anastomoses improved by duct-to-mucosa sutures *Am. J. Surg.*, **161**, 45–50.

[8] Delcore, R., Thomas, J. H., Pierce, G. E. and Hermreck, A. S. (1990). Pancreatogastrostomy: A safe drainage procedure after pancreatoduodenectomy. *Surgery*, **108**, 641–643.

[9] Kapur, B. M. L. (1986). Pancreaticogastrostomy in pancreaticoduodenal resection for ampullary carcinoma: Experience with thirty-one cases. *Surgery*, **100**, 489–493.

[10] Mason, G. R. and Freeark, R. J. (1995). Current experience with pancreatogastrostomy. *Am. J. Surg.*, **169**, 217–219.

[11] Yeo, C. J., Cameron, J. L., Maher, M. M. *et al.* (1995). A prospective randomized trial of pancreaticogastrostomy versus pancreaticojejunostomy after pancreaticoduodenectomy. *Ann. Surg.*, **222**, 580–592.

[12] Buchler, M., Friess, H., Klempa, I. *et al.* (1992). Role of octreotide in the prevention of postoperative complications following pancreatic resection. *Am. J. Surg.*, **163**, 125–131.

[13] Pederzoli, P., Bassi, C., Falconi, M. *et al.* (1994). Efficacy of octreotide in the prevention of complications of elective pancreatic surgery. *Brit. J. Surg.*, **81**, 265–269.

[14] Montorsi, M., Zago, M., Mosca, F. *et al.* (1995). Efficacy of octreotide in the prevention of pancreatic fistula after elective pancreatic resections: A prospective, controlled, randomized clinical trial. *Surgery*, **117**, 26–31.

[15] Friess, H., Beger, H. G., Sulkowski, U. *et al.* (1995). Randomized controlled multicenter study of the prevention of complications by octreotide in patients undergoing surgery for chronic pancreatitis. *Brit. J. Surg.*, **82**, 1270–1273.

Charles J. Yeo, MD
Professor of Surgery and Oncology
The Johns Hopkins Medical Institutions
Baltimore, Maryland, USA

### Budd-Chiari Syndrome: Shunt or Transplant?

### ABSTRACT

*Hemming, A. W., Langer, B., Greig, P., Taylor, B. R. Adams, R. and Heathcote, J. (1996) Treatment of Budd-Chiari syndrome with portosystemic shunt or liver transplantation. The American Journal of Surgery;* **171***: 176–181.*

***Background:*** **Budd-Chiari syndrome is an uncommon disorder caused by obstruction to hepatic venous outflow, causing varying degrees of hepatic injury depending on the extent, severity, and acuity of the obstruction.**

***Patients and Methods:*** **We reviewed the indications for operative intervention and the results of treating 32 patients with Budd-Chiari syndrome seen at Toronto Hospital between 1968 and 1995.**

***Results:*** **Twenty-one patients underwent porto-systemic shunt (PSS) and 7 patients underwent liver transplantation (LT) as their initial operative management. Three patients who initially had PSS subsequently required LT. Patients with cirrhosis found on biopsy and preservation of hepatocellular function were treated with PSS and showed**

**no difference in outcome when compared with patients without cirrhosis (*P*=0.35). Patients who were treated by PSS with retrohepatic vena caval compression, as shown by high caval gradients had outcomes similar to those for patients with low gradients (*P*=0.31). Using the Kaplan-Meier method, 5-year survival of PSS patients was 57%. Liver transplantation was used to manage patients with hepatic decompensation, as well as patients with vena caval occlusion or failed PSS. The 5-year Kaplan-Meier survival for LT was 67%.**

***Conclusions:* Both PSS and LT are effective options in the management of Budd-Chiari syndrome. Portosystemic shunt is the preferred initial approach even with cirrhosis or retrohepatic caval compression as long as there is preservation of liver function and a patent vena cava. Liver transplantation should be used as primary therapy for patients with irreversible hepatic decompensation or vena caval occlusion, and it can be an effective salvage procedure following failed PSS. (*Am. J. Surg.*, 1996; 171, 176–181).**

*Keywords:* Portal hypertension, portacaval shunt, liver transplant

### PAPER DISCUSSION

Budd-Chiari syndrome (BCS) is no longer a rare condition. Since publication of the initial description in 1842, over 2,000 cases have been reported in the medical literature. During the decade from 1981 to 1990, the number of publications cited yearly in *Index Medicus* increased from 32 to 64. The increase in incidence is likely the result of increased awareness of BCS, improvement in diagnostic methods, and widespread use of thrombogenic agents such as oral contraceptives.

During the past two decades, a substantial number of reports of surgical treatment of BCS, similar to the report of Hemming and colleagues, have been published. These reports have consisted mainly of retrospective reviews of case records of patients treated over a long period of time by many surgeons performing a variety of operations in the absence of a well-defined, prospective plan of treatment. It is not possible to perform valid statistical analyses, or base valid conclusions, on such data. Moreover, as in the report of Hemming *et al.*, follow-up has been inconsistent and has not been performed by the surgeons who reported the results. Additionally, comparison of different modalities of therapy such as portosystemic shunt and liver transplantation is not valid when the therapies being compared were performed in different time periods on patients with different stages of liver disease.

Despite these shortcomings, Hemming and associates have drawn some important conclusions, as follows:

1. Portosystemic shunt (PSS) is the preferred initial treatment in BCS without inferior vena cava (IVC) obstruction as long as there is preservation of liver function.
2. Cirrhosis in itself is not a contraindication to PSS if hepatocellular function has been preserved.
3. Synthetic PSS materials should be avoided.
4. IVC compression with high caval gradients is not a contraindication to PSS as long as the IVC remains patent.
5. Following surgical therapy of BCS, patients should be maintained on long-term anticoagulation.
6. Liver transplantaiton (LT) is indicated in (a) patients with cirrhosis and poor hepatocellular function, (b) patients with failed PSS, and (c) patients with fulminant hepatic failure due to BCS.
7. LT is preferred over mesoatrial shunts for patients with IVC obstruction and preservation of hepatocellular function.

We agree wholeheartedly with all of these conclusions except the last one.

We have performed prospective studies of BCS in 41 patients who were treated by portal decompression and then underwent careful follow-up by us that averaged more than six years. Our results have been published [1–5] and are summarized in Table I. In support of the conclusions of Hemming and associates, we have found side-to-side portacaval shunt to be consistently effective in patients with BCS caused by thrombosis of the hepatic veins. In

TABLE I Long-term results of portal decompression operations in 41 patients with BCS treated by Orloff *et al.*

| | Hepatic vein occlusion alone-PCS | IVC and hepatic vein occlusion | |
|---|---|---|---|
| | | Mesoatrial shunt | Combined PCS and CAS |
| No. of patients | 23 | 8 | 10 |
| Onset to operation | | | |
| ≤17 weeks (%) | 83 | 88 | 100 |
| Mean No. of weeks | 17 | 12 | 15 |
| Range weeks | 4–78 | 19 | 10–18 |
| Follow-up (years) | | | |
| Mean | 10.0 | 6.0 | 6.2 |
| Range | 1.0–22 | 2–13 | 1–10 |
| Ascites (%) | 0 | 63 | 0 |
| Need for diuretics (%) | 0 | 63 | 0 |
| Abnormal liver function tests (%) | 14 | 63 | 0 |
| Portasystemic encephalopathy (%) | 0 | 38 | 0 |
| Employed or housekeeping (%) | 95 | 25 | 90 |
| Angiography results (%) | | | |
| Patent shunt | 91 | 37 | 100 |
| Patent IVC | 100 | 0 | 0 |
| Occluded hepatic veins | 100 | 100 | 100 |
| Survival (%) | | | |
| 30-day | 96% | 100 | 100 |
| Current | 91% | 38 | 100 |

our series of 23 patients with hepatic vein occlusion alone, there has been only one operative death (4%) and one long-term death. The 22 survivors of operation have lived free of ascites without diuretic therapy for from 1.0 to 22.0 years (mean 10.0 years). The shunt has remained patent in all but two of the patients, both of whom underwent successful reconstruction of the portocaval anastomosis with a H-graft of autologous internal jugular vein. The revised shunt has remained patent. Liver function has returned to normal in all but three patients who had cirrhosis preoperatively. Hepatosplenomegaly has disappeared, and there has been no encephalopathy. Serial liver biopsies performed 1.0–22.0 years postoperatively showed substantial reversal of the pathologic lesions of BCS. Our experience demonstrates the importance of performing side-to-side portacaval shunt early in the course of BCS in order to reverse the liver damage and prevent extension of thrombosis from the hepatic veins into the inferior vena cava (IVC).

Splenorenal shunt and interposition mesocaval and portacaval shunts using synthetic grafts are hemodynamically similar to side-to-side portacaval shunt but, as Hemming and colleagues point out, are inferior operations in BCS because of a high incidence of thrombosis and occlusion. Interposition shunt using an autologous internal jugular vein H-graft has been used widely in France, and has produced results similar to those of direct side-to-side portacaval shunt.

Side-to-side portacaval shunt is contraindicated when BCS is caused by thrombosis or occlusion of the IVC. Under such circumstances, a mesoatrial shunt with a synthetic graft has been used with some short-term success. However, as pointed out by Hemming *et al.*, there has been a high incidence of thrombosis of synthetic grafts. In our series of eight patients, thrombosis of the mesoatrial shunt developed in five, and the five-year survival rate was only 38%. Similarly, others have reported thrombosis of the mesoatrial shunt in 40–70% of patients, an event that is often followed by death.

Because of dissatisfaction with the results of mesoatrial shunt, we worked in the experimental laboratory to devise a shunting procedure aimed at decompressing the hypertensive IVC and shunting both the portal venous flow and the entire inferior vena caval flow to the right atrium [3]. The new operation, which consisted of a combined side-to-side portacaval shunt and a caval-atrial shunt through a Gore-Tex prosthesis, was found to be very effective in relieving BCS in rats, and has been successful in each of 10 patients with BCS due to IVC occlusion during follow-up of 1.0–10 years (mean of 6.2 years). The combined shunt results in a blood flow rate in the synthetic graft that is much higher than in the mesoatrial shunt. We believe the higher blood flow rate accounts for the absence of thrombosis. In our program, the combined shunt operation has replaced mesoatrial shunt as the preferred treatment for BCS caused by IVC occlusion. We do not agree with the tentative conclusion of Hemming and colleagues that LT is the procedure of choice in patients with IVC obstruction and preservation of hepatocellular function.

Finally, it should be emphasized, as Hemming and colleagues have done appropriately, that PSS and LT are not competing procedures in the treatment of BCS. PSS is the treatment of choice when hepatocellular function is preserved and patent veins are available for accomplishing the shunt. LT is the treatment of choice when hepatic decompensation has developed or when PSS has failed. Both PSS and LT are effective in the treatment of BCS, but they are used for different stages of the disease.

### *References*

[1] Orloff, M. J. and Johansen, K. H. (1978). Treatment of Budd-Chiari syndrome by side-to-side portacaval shunt: experimental and clinical results. *Annals of Surgery*, **188**, 494–512.

[2] Orloff, M. J. and Girard, B. (1989). Long-term results of treatment of Budd-Chiari syndrome by side-to-side portacaval shunt. *Surgery, Gynecology and Obstetrics*, **168**, 33–41.

[3] Orloff, M. J., Daily, P. O. and Girard, B. (1992). Treatment of Budd-Chiari syndrome due to inferior vena cava occlusion by combined portal and vena caval decompression. *Americal Journal of Surgery*, **163**, 137–143.

[4] Orloff, M. J., Orloff, M. S. and Daily, P. O. (1992). Long-term results of treatment of Budd-Chiari syndrome by portal decompression. *Archives of Surgery*, **127**, 1182–1188.

[5] Orloff, M. J. and Orloff, M. S. (1994). Budd-Chiari syndrome and veno-occlusive disease. In: *Surgery of the Liver and Biliary Tract*, 2nd edn., Blumgart, L. H. (Ed.), Churchill-Livingston, London, pp. 1725–1759.

Marshall J. Orloff, MD
Professor of Surgery
University of California, San Diego
Medical Center
200 West Arbor Drive
San Diego, CA 92103-8999

